# Light-harvesting in photosystem I

**DOI:** 10.1007/s11120-013-9838-x

**Published:** 2013-05-04

**Authors:** Roberta Croce, Herbert van Amerongen

**Affiliations:** 1 Department of Physics and Astronomy, Faculty of Sciences, VU University Amsterdam, De Boelelaan 1081, 1081 HV Amsterdam, The Netherlands; 2 Laboratory of Biophysics, Wageningen University, P.O. Box 8128, 6700 ET Wageningen, The Netherlands

**Keywords:** Excitation energy transfer, Picosecond fluorescence, Red forms, Charge separation, State transitions

## Abstract

This review focuses on the light-harvesting properties of photosystem I (PSI) and its LHCI outer antenna. LHCI consists of different chlorophyll *a*/*b* binding proteins called Lhca’s, surrounding the core of PSI. In total, the PSI-LHCI complex of higher plants contains 173 chlorophyll molecules, most of which are there to harvest sunlight energy and to transfer the created excitation energy to the reaction center (RC) where it is used for charge separation. The efficiency of the complex is based on the capacity to deliver this energy to the RC as fast as possible, to minimize energy losses. The performance of PSI in this respect is remarkable: on average it takes around 50 ps for the excitation to reach the RC in plants, without being quenched in the meantime. This means that the internal quantum efficiency is close to 100 % which makes PSI the most efficient energy converter in nature. In this review, we describe the light-harvesting properties of the complex in relation to protein and pigment organization/composition, and we discuss the important parameters that assure its very high quantum efficiency. Excitation energy transfer and trapping in the core and/or Lhcas, as well as in the supercomplexes PSI-LHCI and PSI-LHCI-LHCII are described in detail with the aim of giving an overview of the functional behavior of these complexes.

## Introduction

**Fig. 1:**
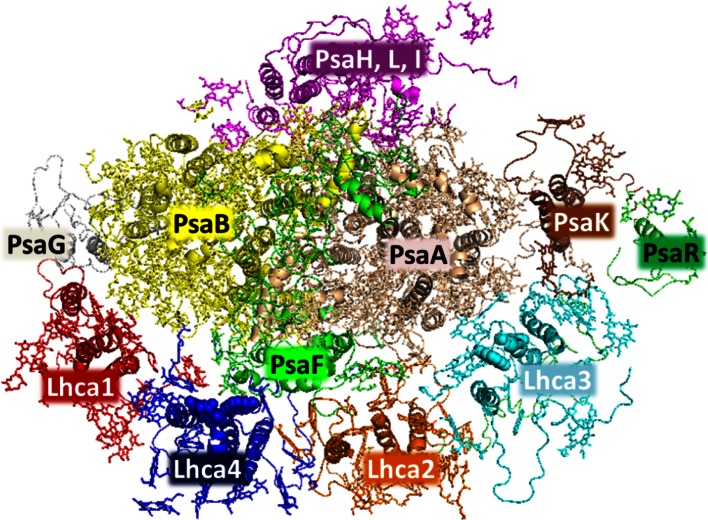
Structure of PSI-LHCI from pea (Amunts et al. 2010). *Top view* from the stromal side. The main subunits of core and antenna are indicated in figure. The Chls responsible for the red forms in Lhca4 and Lhca3 are presented in space-filled style

Photosystem I (PSI) is the multiprotein complex that reduces ferredoxin and oxidizes plastocyanin. It is composed of a core complex which contains around 100 chlorophylls *a* (Chls *a*) and all the cofactors of the electron transport chain and in most cases of an outer antenna system that increases the light-harvesting capacity. The core complex is conserved in all organisms performing oxygenic photosynthesis, while the outer antenna varies for different organisms. In plants, it is composed of Chl *a* and *b* binding proteins (Lhca’s) belonging to the light-harvesting complex (Lhc) multigenic family and together they are called LHCI. In total, the PSI-LHCI complex of higher plants coordinates around 170 Chl molecules and 30 carotenoid molecules. In high-light conditions (2,000 μE/m^2^s), this complex absorbs on average one photon per 600 μs. The structure of the PSI-LHCI complex of pea in which four Lhca’s are associated with the core complex, is presented in Fig. 1. Structural details about the complexes can be found in Jordan et al. (2001) and Amunts et al. (2010), while the present review focuses on the light-harvesting process and the high energy conversion efficiency of this complex.

### The basis of the high quantum efficiency of PSI

Photosystem I is known to be the most efficient light converter in nature (Nelson 2009), with a quantum efficiency (defined as the number of electrons produced per number of absorbed photons) that is close to 1. This fact is even more amazing, if we consider that PSI in plants contains around 200 pigments (Amunts et al. 2010). To achieve this high efficiency, it is necessary (1) that the energy is transferred very rapidly to the primary (electron) donor, (2) that the pigments in the complex are not being quenched, and (3) that the charge separation is to a large extent irreversible. In general, the published kinetic results on excitation trapping can be and have been modeled in different ways (see below), but all models have these three properties incorporated. In this review, we will mainly focus on excitation energy transfer (EET) and pay less attention to the charge-transfer processes. For the latter, we refer to an excellent review by Savikhin (2006).

The rate of energy transfer depends strongly on the distance between donor and acceptor of the excitation energy: the closer the pigments are the faster the transfer goes (although the mutual orientation also plays a role) and the higher the efficiency will be (Forster 1948). Down to a mutual center-to-center distance *R* between pigments of 1.5 nm, the transfer rate scales with *R* ^−6^ according to the Förster equation whereas as shorter distances excitonic effects start to play a major role and excitations start to become more and more delocalized over the different pigments (see, e.g., van Amerongen et al. (2000)). However, if the pigments are getting too close, then an unwanted secondary effect called concentration quenching may occur, leading to a shortening of the excited-state lifetime, thereby decreasing the quantum efficiency (Beddard and Porter 1976). Very roughly, PSI of plants can be approximated by a cylinder of 12-nm diameter and 5-nm height, containing 170 Chls. This means that the pigment concentration in this system is 0.5 M. The excited-state lifetime of a diluted solution of Chls is around 6 ns, but it is below 100 ps at 0.5 M in lipid vesicles (Beddard et al. 1976). Apparently, PSI is able to avoid concentration quenching to keep the quantum efficiency close to 1.

What is the trick? It is the protein that keeps the pigments at the correct distance and geometry to facilitate fast energy transfer and to prevent excited-state quenching. In addition, the protein has a role in tuning the energy levels of the pigments (defining at which wavelength/color the maximum absorption occurs) whereas its vibrations (phonons) can couple to the electronic transitions of the pigments to broaden the absorption spectra and to allow energy transfer (both uphill and downhill) through the excited-state energy landscape (Van Amerongen et al. 2000).

But this is not yet all. When one reads about the energy transfer efficiency, it is nearly always written that EET should follow an energy gradient (from high-energy pigments to low-energy ones) to be efficient. Indeed, the picture used to exemplify photosynthetic energy transfer is commonly a deep funnel, where the energy is transferred between pigments of colors throughout the whole rainbow to end up on the primary donor which is the pigment with the lowest excited-state energy. This picture fits rather well with the antennae of cyanobacteria, the phycobilisomes, but it is clearly not a realistic representation of the situation in plants and green algae in which the most of the pigments are more or less isoenergetic. While it is correct for PSI that the primary electron donor (absorbing around 700 nm) is lower in energy than the bulk pigments (the maximum absorption of PSI is at 680 nm), it is also true that almost all PSI complexes contain Chls that absorb at energies below that of the primary donor, and they are responsible for the so-called red forms (Karapetyan 2006; Brecht et al. 2009). It was already shown in Croce et al. (1996) that at physiological temperatures a large part of the harvested energy reaches the red forms and thus needs to be transferred against the energy gradient in order to be used for charge separation. This is possible at the physiological temperatures at which these organisms live because thermal energy fills the energetic gap between donor and acceptor (Jennings et al. 2003). This means that the energy transfer pathways in PSI should be pictured more like a track for a roller coaster than like a descending road. Despite the presence of these pseudo traps, the system is extremely efficient. The role of these red forms in plants has not been completely elucidated yet, although it is clear that they extend the absorption capacity of the system to harvest solar energy in the near infrared, and thus provide an advantage in canopy or dense culture situations where the visible light is efficiently absorbed by the upper levels of the cells (Rivadossi et al. 2003). It has also been proposed that the red forms are important in photoprotection (Carbonera et al. 2005), and that they concentrate the excitation energy close to the reaction center (RC) (Trissl 1993). Although it should be mentioned that there are also red forms far away from the RC, and for example, the most red forms in plants are associated with LHCI (Croce et al. 1998). In the case of cyanobacteria, the red forms have a dual role which depends on the redox state of PSI: Karapetyan et al. (1999, 2006) and Schlodder et al. (2005) have shown with *Arthrospira platensis* that when the PSI RC is open, the energy absorbed by the red Chls migrates uphill to P700 at physiological temperatures thus increasing the absorption crosssection. If the PSI RC is closed, then the energy absorbed by the red Chls is dissipated, thus preventing PSI photodamage. The difference between plants and cyanobacteria is largely due to the location of the red forms: in higher plants, the red forms are mainly associated with the outer antenna (Croce et al.^1998^) and are distant from P700, while the red forms in the cyanobacterial core are supposed to be rather close to P700. This is supported by the observation that there is no energy transfer from LHCI to P700 in PSI of higher plants and algae at cryogenic temperatures, while energy migration from red Chls to P700 in PSI of cyanobacteria takes place even at cryogenic temperatures (Karapetyan 2006).

In the following, we will first describe the light-harvesting properties of the core and of the individual antenna complexes of higher plants before to move to the PSI-LHCI and PSI-LHCI-LHCII supercomplexes. A large part of the available data regarding the core complex has been obtained on cyanobacterial cores, and will only be briefly summarized here. Regarding LHCI and PSI-LHCI complexes, those of plants are clearly the best-studied ones, and the review will mainly focus on them. However, during recent years, information about PSI organization/composition/function of other eukaryotic organisms has emerged and the last paragraph focuses on the complex of *Chlamydomonas reinhardtii*, which is at present the best-studied alga system.

#### The core complex

**Fig. 2:**
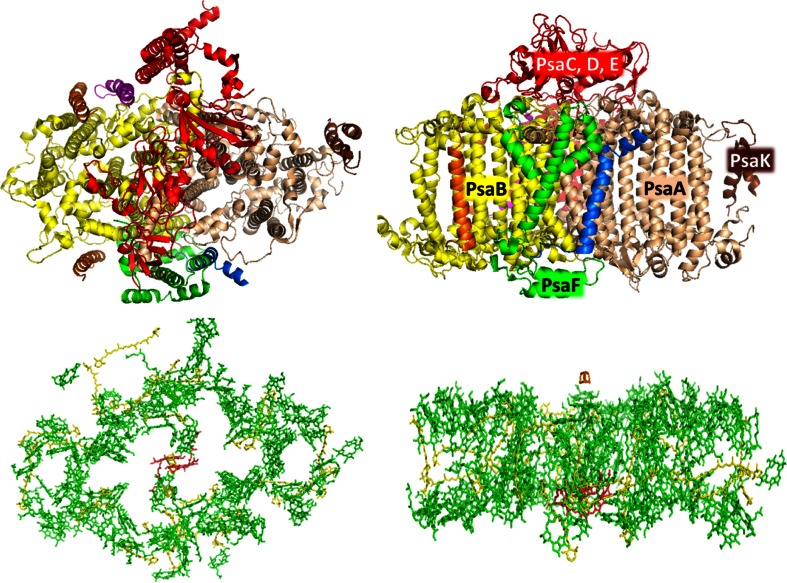
Structure of the cyanobacteria core (Jordan et al. 2001). *Top* protein organization. *Left*, *top view* from the stomal side. *Right*, *side view* the main proteins are indicated in figure, the color code for *left* and *right* is identical. *Bottom* pigment organization. Chlorophylls are in *green* with the exception of P700 which is in *red*. Carotenoids are in *yellow*. *Left* and *right* as in the *top panel*

The core complex of PSI (Fig. 2) is composed of 11–14 subunits depending on the organism, and it coordinates 96 Chls *a* and 22 β-carotene molecules in cyanobacteria (Fromme et al. 2001; Amunts et al. 2010). The main difference between PSI in cyanobacteria and higher plants is that the former occurs as a trimer, and the second one as a monomer. The pigments are mainly associated with the two largest subunits PsaA and PsaB, while the small subunits bind only a few Chls. For a detailed overview of the properties of the core subunits, the reader is referred to Jensen et al. (2007). The primary donor of PSI (P700) absorbs around 700 nm, below the energy of the bulk chlorophylls with average absorption around 680 nm. Nearly all PSI complexes also contain red forms (Karapetyan et al. 1999), but while in cyanobacteria the most red forms are associated with the core, in higher plants they are present in the outer antenna (Croce et al. 1998). The presence of red forms in the higher plant core is at present a point of discussion (Slavov et al. 2008). The absorption/emission of these forms varies for different organisms with emission maxima ranging from 720 to 760 nm (Gobets and van Grondelle 2001; Karapetyan 1998). Their number also varies and they are responsible for 3–10 % of the absorption in the region above 630 nm. Although it has been suggested that these forms originate from strongly interacting Chls (e.g., Gobets et al. 1994; Zazubovich et al. 2002), and several candidate pigments have been put forward (Zazubovich et al. 2002; Sener et al. 2002; Byrdin et al. 2002), it is still not exactly known which Chls are responsible for these forms. More in general, it should be noticed that all pigments in the core are very close together (see Fig. 2 bottom; average center-to-center distance between neighbors is around 10 Å), facilitating very efficient energy transfer. Indeed, many of the transfer steps between neighboring pigments were observed to take place with time constants between 100 and 200 fs (Du et al. 1993). The energy transfer to the red forms is slower and occurs in around 2–10 ps depending on the number of red forms in the different organisms (Savikhin et al. 2000; Hastings et al. 1995; Melkozernov et al. 2000a; Gobets and van Grondelle 2001; Gibasiewicz et al. 2001; Muller et al. 2003). This makes sense of course because there are only a few Chls responsible for this red-shifted absorption and many transfer steps are needed to reach them. It was shown that energy transfer and trapping in practically all PSI core complexes can be described with the same model which is composed of two parts: One part which represents the transfer from the bulk Chls to the primary donor and which is identical for all PSI species and other that depends on the different red-form contents and energy levels and thus is species-dependent. It was shown that the overall trapping time (the average time between the moment of absorption of a photon by one of the Chls in the core and the moment of charge separation) in the absence of red forms is around 18 ps (Gobets et al. 2001b). The presence of low-energy Chls slows down the trapping time; how much exactly depends on the number of red forms as mentioned above, but also on their excited-state energy levels: the more red forms there are and the lower their energy is, the longer it takes to transfer the excitations back from these Chls to pigments with higher energy, which is needed to reach the RC. For a comprehensive study in which different complexes were compared, we refer to Gobets et al. (2001b).

In summary, EET and trapping in the PSI core are very fast (20–40 ps), which means that the complex is very efficient in using sunlight despite the presence of chlorophylls that absorb at energies lower than the primary electron donor in the RC and partially slow down the EET. However, these red forms also broaden the absorption spectrum, apparently increasing the light-harvesting capacity.

##### Is charge separation in PS migration-limited or trap-limited?

There is a long-standing discussion whether the excitation energy trapping (i.e., the disappearance of an excitation due to charge separation) in the core of PSI is trap-limited, migration-limited (also called diffusion-limited) or something in between. If charge separation is migration-limited, then this means that the overall trapping time is dominated by the time it takes for an excitation to reach the primary donor P700 after which charge separation is so fast that the excitation cannot escape anymore into the antenna. On the other hand, when charge separation is trap-limited, EET is extremely fast, and an excitation might visit P700 many times before it gets trapped. However, experimentally it is very difficult to determine which model is the most appropriate for the core of PSI. Ultrafast fluorescence and transient absorption measurements have demonstrated that spectral equilibration occurs very rapidly, which at first sight may seem to argue against a migration-limited model. Savikhin et al. (2000) for instance observed spectral equilibration times of 0.53 and 2.3 ps, followed by charge separation from a spectrally equilibrated core with a time constant of 23.6 ps. However, it should be realized that spectral equilibration and spatial equilibration are not the same thing. It is very well possible that rapid spectral and spatial equilibration occur in the antenna surrounding the RC (see also below), but that subsequent EET to the primary donor is relatively slow, which corresponds to the so-called transfer-to-the-trap-limited model that was advocated in various studies (Gobets and van Grondelle 2001; Gobets et al. 2001b). However, as was pointed out by Savikhin (2006), it is often extremely difficult (if not impossible) to conclude from the experimental data alone, which model is correct. The main reason for adopting the transfer-to-the-trap-limited model is that the average distance between neighboring pigments in the surrounding antenna is much shorter than between the RC pigments and the antenna pigments. Although it is true that there are some “linker” pigments between RC and antenna, there are only two of them, one on each side of the RC, whereas most antenna pigments have several neighbors very close by. In an illustrative modeling study by Gobets et al. (2003), the distances between pigments were explicitly taken into account. Use was made of the Förster equation for calculating interpigment EET to explain the overall trapping time of 18 ps in the absence of red forms. It was found that when an average hopping time of 150 fs (average lifetime of an excitation on a single pigment) was taken, right in the middle of the interval 100–200 fs mentioned before, a value of ~9 ps was found for the delivery time of an excitation to the primary donor. However, in that case a value of *n* = 1.21 is needed for the refractive index in the Förster equation to get a consistent description of the data, and this value seems rather low (Knox and van Amerongen 2002), although it has also been argued that for closely spaced pigments in PSI this may not be unrealistic (Damjanovic et al. 2002; Yang et al. 2003). Byrdin et al. (2002) used an approach where excitonic interactions were included to get a rather good description of the absorption, linear-dichroism, and circular-dichroism spectra of PSI from *Thermosynechococcus elongatus*. In order to get such a description, variations in the excited-state energy levels (site energies) of individual Chls were required and a certain assignment was chosen that led to the rather good simulated spectra. This assignment is certainly not unique, but the influence of variation of the site energies can be tested (see also below). For the energy-transfer calculations a hybrid approach was used, where transfer rates between pairs of pigments were calculated with the use of the Förster equation like Gobets and coworkers did but for each pair of pigments a weighted average was taken over the different exciton states in which the Chls were participating. The best description was obtained for an intrinsic charge-separation time of 0.9 ps^−1^, and concomitantly, the charge-separation process was neither pure trap-limited nor transfer (-to-the-trap)-limited. More recently (Adolphs et al. 2010), the absorption, circular-dichroism, and linear-dichroism spectra were obtained with quantum-chemical/electrostatic calculations, i.e., with methods that had proven to lead to more or less correct site energies for simpler systems like the FMO complex and the calculated spectra showed rather good agreement with the experimental spectra. The results have not been used yet for describing the energy transfer properties, but it was concluded that the concentration of low-energy exciton states in the antenna is larger on one side of the RC, implying asymmetric delivery of excitation energy to the RC (Adolphs et al. 2010). The authors also proposed experiments to verify their calculations/predictions. Sener et al. (2002) also simulated EET transfer in PSI from *Thermosynechococcus elongatus* using a Förster-type approach and concluded that the overall transfer process does not depend very much on room-temperature fluctuations of the site energies, which were all chosen to fluctuate around a common average value. Damjanovic et al. (2002) performed quantum-chemical calculations that showed substantial variations of the site energies of the Chls in PSI, leading to an overall absorption spectrum that was in reasonable agreement with the experimental one. However, these values did not lead to substantial changes in the overall diffusion time of excitations according to Sener et al. (2002). A very insightful modeling study is the one of Yang et al. (2003) in which excitonic interactions are not only used to calculate steady-state spectroscopic properties but are also included to model the excitation dynamics. The authors find that spectral and spatial equilibration outside the RC both occur within 5 ps, whereas the excitation transfer to the primary donor P700 is responsible for the largest contribution to the trapping time. Omitting the linker pigments in the simulations leads to somewhat slower transfer to the RC, but the overall trapping time is not changed substantially. Interestingly, the transfer from the antenna to P700 proceeds to a large extent via the other Chls in the RC and omitting those from the simulations slows down the transfer to P700 considerably. It is concluded that the combination of linker and RC pigments form a quasi-funnel structure that is highly optimized for efficient trapping. This trapping process is preceded by ultrafast “equilibration” in the antenna (within 5 ps), leading to a so-called transfer equilibrium state, and is followed by charge separation with a time constant between 0.9 and 1.7 ps. However, the actual value of the latter time constant does not influence the overall trapping time to a large extent, in contrast to the situation in trap-limited models. It should, however, be mentioned that not everyone agrees with these results; Muller et al. (2003) have for instance presented a transient absorption study in which it was concluded that charge separation in PSI with red forms is trap-limited. However, we are not aware of any theoretical studies so far that have been able to support this conclusion. On the other hand, it is also interesting to note that Holzwarth and coworkers have found indications that P700 might not be the primary electron donor (Holzwarth et al. 2006) and later work is in agreement with this proposal (Giera et al. 2010). Given the results of the calculations of Yang et al. this would imply that excitations reach the primary donor faster than was thought before. Finally, it is interesting to mention that recently ultrafast charge separation was observed with a time constant below 100 fs when photosystem I from *Synechocystis* was excited with spectrally broad 20 fs laser pulses centered at 720 nm. This is the fastest charge separation reported so far, and it does definitely not support a trap-limited scenario (Shelaev et al. 2010).

In conclusion, it seems most plausible that EET in the antenna system of the core occurs within a few ps (~5 ps) and is followed by far slower transfer to P700 (~20 ps) where charge separation occurs with an electron transfer time of ~1 ps. Although it seemed to be clear for a long time that P700 is the primary electron donor, this is not so certain anymore, meaning that transfer to the primary donor might be faster than was thought before.

#### The antenna complexes of PSI in higher plants

##### Biochemical and spectroscopic properties

A full characterization of the biochemical and spectroscopic properties of native Lhca complexes of *Arabidopsis thaliana*, which are present as functional dimers can be found in Wientjes and Croce (2011). The presence of an outer antenna system associated with PSI core in plants was first reported by Mullet et al. (1980). The first purification of LHCI complexes stems from 1983 by Haworth et al. (1983), who obtained an isolated fraction containing four polypeptides with molecular weights between 20 and 24 kDa. The four Lhca’s correspond to the products of the Lhca1-4 genes. Two more Lhca genes were identified in the genome of *Arabidopsis thaliana*, Lhca5 and 6, but their expression level is always very low in all conditions tested (Ganeteg et al. 2004).

**Fig. 3:**
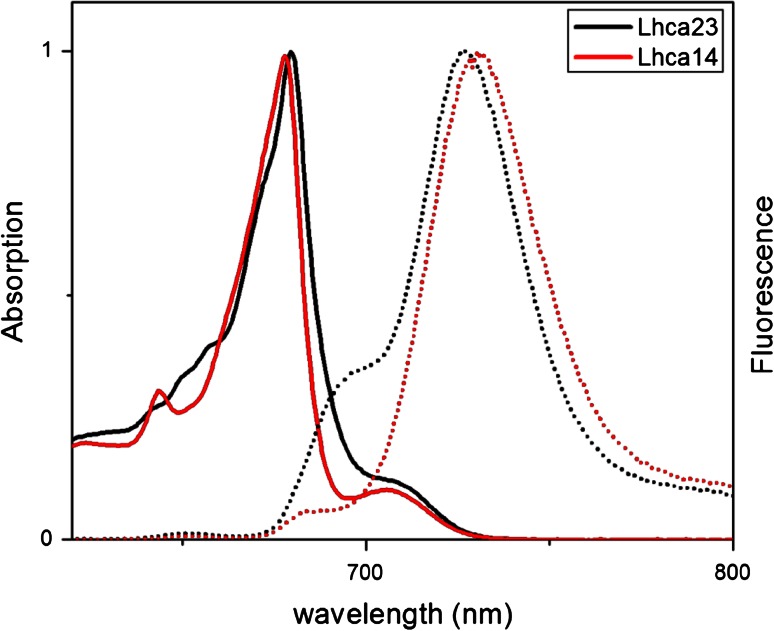
Absorption (*solid*) and fluorescence emission (*dot*) spectra of Lhca1/4 (*red*) and Lhca2/3 (*black*) native dimers at 77 K (Wientjes et al. 2011a)

For a long time, it was believed that the LHCI antenna is composed of two complexes, called LHCI-730 and LHCI-680 based on their emission properties, with the former being enriched in Lhca1–Lhca4 and the latter in Lhca2 and Lhca3 (Lam et al. 1984; Bassi et al. 1985). However, while the properties of the Lhca1-4 heterodimer were studied on isolated and reconstituted complexes (Schmid et al. 1997; Knoetzel et al. 1992; Tjus et al. 1995; Croce et al. 2002), questions remained about the properties and the aggregation state of Lhca2 and Lhca3 due to the impossibility to purify them to homogeneity or even to reconstitute the dimer in vitro. Only recently all Lhcas were purified as two functional heterodimers, Lhca1/4 and Lhca2/3 (Wientjes and Croce 2011). They both emit in the red, with a maximum around 730 nm at low temperature. The absorption and emission spectra of the native dimers are reported in Fig. 3. This result is the final proof that the LHCI-680 complex that for a long time was believed to be part of the antenna of PSI is simply the result of a partial denaturation of the complexes with a loss of red forms: LHCI-680 complex does not exist in vivo.

The X-ray structure of the PSI-LHCI complex shows that each Lhca binds 13–14 Chls molecules (Ben-Shem et al. 2003), and the biochemical data indicate for both dimers a Chl *a*/*b* ratio of 3.7, meaning that they have lower affinity for Chl *b* than the complexes of PSII (LHCII has a Chl *a*/*b* ratio of 1.33). The dimers also bind five carotenoids each, mainly lutein and violaxanthin and substoichiometric amounts of β-carotene, while neoxanthin is not present at all, at variance with the antenna of PSII (Wientjes and Croce 2011).

The properties of the individual Lhca’s have been studied by in vitro reconstitution of the complexes of tomato and *A. thaliana* (Schmid et al. 1997, 2002; Croce et al. 2002; Castelletti et al. 2003) because at present it is still not possible to obtain native preparations of pure Lhca monomers. The Lhca’s seem to be stable in their dimeric form, while monomerization leads to the loss of some pigments. However, the properties of the reconstituted monomers were shown to be in agreement with the properties of the native dimers (Wientjes and Croce 2011). Although the properties of all individual monomers differ substantially from each other, it is interesting to notice that many spectral and biochemical properties of the dimer Lhca1+4 are very similar to those of Lhca2+3. For example, Chl *a*/*b* is 3.7 for both dimers whereas the Chl *a*/*b* ratios are 4.0 for Lhca1, 6.2 for Lhca3, 1.85 for Lhca2, and 2.3 for Lhca4 (Castelletti et al. 2003). Although the general structure and pigment coordination of Lhca complexes are very similar to those of the Lhcb antennae, which are mainly associated with PSII, Lhcas differ from Lhcbs because of the presence of low-energy absorption forms. The corresponding electronic transitions are responsible for fluorescence emission that is 50 nm red-shifted as compared to the emission of Lhcb complexes. Lam et al. (1984) observed for the first time emission of a purified fraction containing LHCI complexes that was peaking around 730 nm at 77 K, indicating that at least one of the complexes should contain red forms. The first candidate was Lhca4 (Bossmann et al. 1997; Zhang et al. 1997; Schmid et al. 1997) as suggested both by the analysis of plants lacking individual complexes and by in vitro reconstitution. Later it was shown that also Lhca3 emits above 725 nm and that Lhca1 and Lhca2 emit at 690 and 702 nm (Ganeteg et al. 2001; Croce et al. 2002; Schmid et al. 2002; Castelletti et al. 2003). This means that all Lhca’s emit at energies below those of the antenna of PSII (680 nm). Lhca5 does not contain red forms and emits at 684 nm (Storf et al. 2005). Nothing is known about Lhca6, which has, however, a primary structure that is very similar to that of Lhca2 and probably has also similar spectroscopic properties.

The origin of the red forms in the Lhca complexes of higher plants was studied by mutation analysis and in vitro reconstitution (Morosinotto et al. 2002, 2005b; Croce et al. 2004; Mozzo et al. 2006). It was shown that the Chls that are responsible for the low-energy absorption in all Lhca’s are Chls 603 and 609 (nomenclature from Liu et al. (2004), A5 and B5 according to Kuhlbrandt et al. (1994), these Chls are represented in space-fill style in Fig. 1), and that the difference in energy between the lowest energy state of the four complexes is due to variation in the interaction strength between these Chls. In Lhca3 and Lhca4 that harbor the most red forms, the ligand for Chl 603 is an asparagine, and it was shown that this residue is essential for stabilizing the most red form (Morosinotto et al. 2003). It was suggested that the presence of this asparagine maintains the correct geometry between the interacting Chls allowing for the formation of a charge-transfer (CT) state (Croce et al. 2007; Romero et al. 2009). More recently, a correlation between the presence of the asparagine as ligand for Chl 603 and the most red forms was also observed for the complexes of *Chlamydomonas reinhardtii* (Mozzo et al. 2010) and *Physcomitrella patens* (Alboresi et al. 2011 *)*, but it was suggested that this might not be the case in *Ostreococcus tauri* (Swingley et al. 2010). We would like to stress once more that the asparagine per se is not responsible for the red forms (and thus that the presence of an asparagine as ligand for a Chl is not a condition sufficient to induce red absorption), but Asn is necessary for maintaining the right geometry between the interacting Chls in the Lhca protein to allow for strong interaction, which is the reason for the red shift. Stark spectroscopy has shown that the red forms of Lhca4 originate from the mixing of the lowest excited state of a strongly coupled Chl dimer and a CT state (Romero et al. 2009), supporting earlier suggestions about the origin of these forms in Lhca complexes (Ihalainen et al. 2003) and in the core (Zazubovich et al. 2002; Vaitekonis et al. 2005).

In summary, four Lhca complexes (Lhca1–4), organized in two dimers (Lhca1–4, Lhca2–3), compose the outer antenna system of PSI in plants. The biochemical and spectroscopic properties of the dimers are very similar, and they both contain red forms (fluorescence maximum around 730 nm at 77 K) that originate from the mixing of the lowest excitonic state of a chlorophyll dimer (603(A5)/609(B5)) and a CT state.

##### Excitation energy transfer

Excitation energy transfer has been studied in reconstituted Lhca1 and Lhca4 and the native dimers of *Zea mays*, *A. thaliana*, and tomato (Melkozernov et al. 1998, 2000b, 2002; Gobets et al. 2001a; Gibasiewicz et al. 2005a; Wientjes et al. 2011a). It was shown that the equilibration in the Lhca4 monomeric complex occurs in <5 ps with EET from Chls *b* to Chls *a* occurring with time constants of 300 fs and 3 ps. All energy transfer processes, including carotenoid to Chl EET, seem to occur on a similar time scale as observed for the antenna complexes of PSII, as one might expect from the similar structure and pigment organization (Gibasiewicz et al. 2005a). The major difference is the fact that the lowest energy state is located on Chls 603/609 (instead of on Chls 610/611/612 (A1/B2/A2). The analysis of the Lhca1/4 and Lhca2/3 dimers shows that the transfer between monomers in the dimers is slower than the equilibration within a monomer, and it occurs in around 12 ps (Wientjes et al. 2011a). The average excited-state lifetime of the native complexes is 2.7 ns (Wientjes et al. 2011a), while shorter values were observed for the recombinant complexes (Melkozernov et al. 2000b; Ihalainen et al. 2005a; Passarini et al. 2010). The fluorescence decay is multiexponential for monomers and dimers, suggesting the presence of different conformations (Moya et al. 2001). The decay kinetics of Lhca complexes can be described with four components with lifetimes between 300 ps and 4 ns; the shortest component shows a spectrum with maximum at 690 nm and the longest one as a maximum at 720 nm (Wientjes et al. 2011a; Passarini et al. 2010). These components correspond to different protein conformations as shown by single-molecule spectroscopy (Kruger et al. 2011). The equilibrium between the conformations can be changed by mutating particular residues in the proximity of the two interacting Chls responsible for the red forms: Small changes in the structure (e.g., the substitution of the asparagine by a glutamine) lead to a complete change in the equilibrium between the conformations (Wientjes et al. 2012). This means that they can easily switch from a “light-harvesting” state to a “quenched” state.

This property seems to be common to all members of the Lhc multigenic family (Moya et al. 2001; Kruger et al. 2011). Indeed, the light-harvesting complexes have been suggested to be involved in light-harvesting as well as in photoprotection (Ruban and Horton 1995). This means that they are able to optimize the absorption of photons and the transfer of excitation energy to the RC to maintain a very high quantum efficiency, but they are also able, when necessary, to quench their excited states and dissipate the excess energy as heat, thus preventing photodamage. When the Lhca’s are connected to the core, they probably exist in their “light-harvesting” state to maximize the use of sunlight. One might speculate that the capacity of changing conformation becomes important when the antenna complexes are disconnected from the core and need to lower their excited-state population to minimize triplet formation which can lead to deleterious singlet oxygen formation, which can damage proteins, pigments, and lipids (Krieger-Liszkay et al. 2008). This idea seems to be supported by the fact that monomers (which have more structural degrees of freedom) are more often in the quenched conformation than dimers (Wientjes et al. 2011a; Passarini et al. 2010).

In conclusion, energy equilibration in monomeric Lhca complexes is very fast (5 ps) and occurs before equilibration between both monomers in a dimer. The complexes can exist in different conformations associated with different lifetimes and spectra.

#### PSI-LHCI supercomplex

##### Biochemical and structural characterization

In the PSI-LHCI supercomplex 4 Lhca’s are associated with the core forming half a ring on the side of PsaF/J (Boekema et al. 2001; Ben-Shem et al. 2003; Amunts et al. 2010). It is now generally accepted that one copy each of Lhca1-4 is present per supercomplex (Ballottari et al. 2004) and that each Lhca occupies a fixed position in the structure: The sequence going from the G pole (position of PsaG) of the core to that of K (position of PsaK) (Fig. 1), is Lhca1, Lhca4, Lhca2, and Lhca3 (Amunts et al. 2007; Wientjes et al. 2009). The composition of the outer antenna was found to be constant in all light conditions (Ballottari et al. 2007) and even in mutants lacking individual subunits, the place of the missing complex is not taken by any other Lhca (Klimmek et al. 2005; Morosinotto et al. 2005a; Wientjes et al. 2009), clearly indicating that the complexes are not interchangeable. The only exception is Lhca4 that in the Lhca4 KO mutant is partially substituted by Lhca5 (Wientjes et al. 2009) in agreement with the fact that in vitro Lhca5 is able to form a stable dimer with Lhca1 (Storf et al. 2005). This lowers the content of red forms in the complex as Lhca4 contains red forms, while Lhca5 does not, and may be of importance in specific light conditions. It has also been proposed that Lhca5 is interacting with Lhca2 and Lhca3 (Lucinski et al. 2006) and that Lhca5 and Lhca6 are necessary for the formation of the NADPH dehydrogenase-PSI supercomplex in *A. thaliana* (Peng et al. 2009). Although information about Lhca5 and Lhca6 is still lacking, their low expression levels in all tested conditions indicate that the basic PSI-LHCI unit in higher plants is only composed of the core complex and one copy each of Lhca1-4. The 3D structure has also shown that the PSI-LHCI supercomplex coordinates 173 Chl molecules in total. Around 100 of them are associated with the core as in cyanobacteria, 56 are associated with the Lhca complexes and the others are located in between the Lhca’s and the core and are named “gap” pigments (Amunts et al. 2010). Interestingly, although the structure does not show tight protein–protein interactions between the subunits of the core and the outer antenna, their association appears to be very strong in plants at variance with the association of LHCII to the PSII core, which is rather weak (Wientjes et al. 2009).

In summary, the PSI-LHCI complex in plants is composed of the core plus 4 Lhca’s. The number and organization of the Lhca’s are identical in all growth conditions. The complex contains 173 Chls, most of which are associated with the core and the Lhcas, but a fraction is also located in between them.

##### Excitation energy transfer

A number of studies have investigated the light-harvesting process in the PSI-LHCI supercomplex of plants (Turconi et al. 1994; Croce et al. 2000; Ihalainen et al. 2002; Engelmann et al. 2006; Slavov et al. 2008; van Oort et al. 2008; Wientjes et al. 2011b). All measurements are characterized by the presence of two or three decay components. A fast component <10 ps represents excitation equilibration between the bulk pigments and the red most forms. A decay component in the range of 18–24 ps is normally considered to be associated with direct trapping in the core and a longer component of around 60–100 ps is thought to be due to trapping following excitation in the LHCI complexes. The average lifetime is similar to what was obtained by modeling (Sener et al. 2005). In order to extract details from the time-resolved measurements mainly two methods have been used. Target analysis, in which the complex is divided into several compartments, inside which the equilibration is considered to be very fast. The model fits the time-resolved data, while extracting the rate constants for energy transfer between the compartments. The spectra of the compartments are the second type of output from the fitting and should allow judging the quality of the fitting as they should match the steady-state emission spectra of the different PSI subcomplexes. This method has been used in Slavov et al. (2008). The other possibility is to analyze PSI complexes with different antenna size (Ihalainen et al. 2005b) and to excite at different wavelengths to vary the amount of excitation in the core and in the antenna. This method was used more recently (Wientjes et al. 2011b), measuring PSI-core, PSI-Lhca1/4, and PSI-Lhca1/4-Lhca2/3 upon excitation at 440 nm, which is more selective for the core and at 475 nm which excites preferentially the outer antenna complexes (because they contain Chl *b*, the Soret band of which is around 475 nm). In principle, both methods have their own pro’s and contra’s, but in the end they should lead to the same result. Unfortunately, the analysis of Slavov et al. was done before the Lhca2/3 dimer was fully characterized (Wientjes and Croce 2011), and thus the authors did not have the proper target spectra to validate their model. It would be very interesting to repeat the target analysis now that the spectra are available. In the following, we will summarize the results of Wientjes et al. (2011b), which represent the most recent PSI model, and put forward the points that still need clarification.

**Fig. 4:**
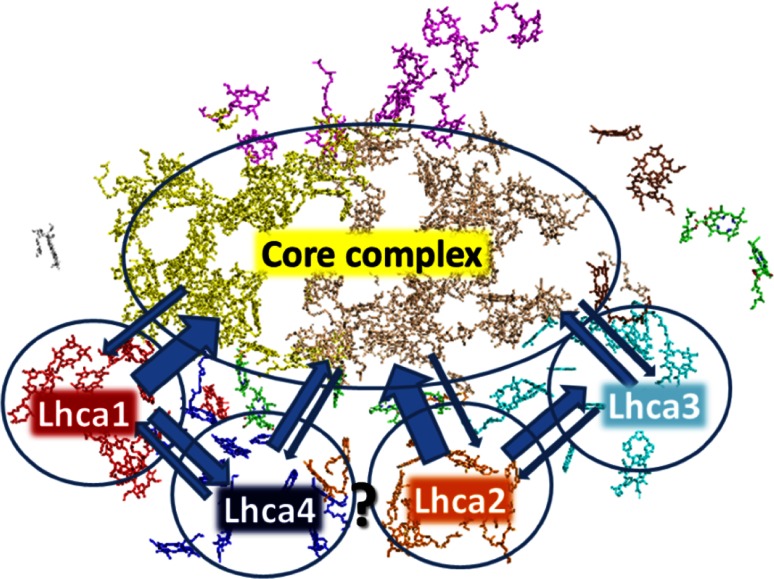
Schematic presentations of energy transfer and trapping in PSI-LHCI based on Wientjes et al. (2011b). Increasing thickness of the *arrows* indicates increasing rates. The transfer rate between Lhca2 and Lhca4 could not be estimated from the target analysis in that study, but based on structural data, it has been suggested to be similar to the intradimer transfer rates

Wientjes et al. observed that all Lhca’s are transferring excitations directly to the core. The transfer from Lhca1 and Lhca2 (here named “blue” complexes) to the core is very fast and occurs in around 10 ps. These two complexes also transfer to the “red” Lhca’s (Lhca3 and Lhca4) with a similar transfer rate. Lhca3 and Lhca4 transfer directly to the core but slower, in around 40 ps. This difference is due to the presence of the red most forms which are lower in energy than the accepting molecule in the core, thereby slowing down the transfer because of what we might call the “roller coaster” effect. It appears that in the end all Lhca’s transfer a similar amount of excitations to the core (Wientjes et al. 2011b). To directly check the influence of the red forms on the trapping time, Wientjes et al. also measured a PSI-LHCI complex which is identical to that of the WT but in which Lhca4 had been substituted with Lhca5 that does not contain red forms. The fastest decay component becomes slower in the presence of Lhca5 (it goes from 20 to 26 ps), but the corresponding amplitude is strongly increased as compared to WT PSI (with Lhca4), whereas the amplitude of the slow component, which corresponds to a red spectrum, has concomitantly decreased. This clearly indicates that the transfer from the “blue” antenna Lhca5 to the core is extremely fast. This experiment also shows that the fast decay component commonly seen in the EET measurements of PSI, is not only due to the trapping from the core, but also from the “blue” antennae. The slow decay originates from Lhca4 and Lhca3. The data show that these red forms together slow down the transfer by a factor of two, in agreement with previous suggestions (Engelmann et al. 2006; Slavov et al. 2008). A scheme of the energy transfer in PSI-LHCI based on Wientjes et al. (2011b) is shown in Fig. 4.

In conclusion, PSI-LHCI in plants the trapping time is around 50 ps. The most red forms are associated with the outer antenna. All Lhca’s transfer excitation energy to the core, the blue Lhca’s (1 and 2) very rapidly and the red ones (Lhca3 and 4) somewhat slower.

#### PSI-LHCI-LHCII supercomplex

In all conditions in which PSII is preferentially excited, part of the LHCII population moves to PSI to increase its antenna size, forming the PSI-LHCI-LHCII supercomplex (e.g., Lemeille and Rochaix 2010). This is considered to be a short-term acclimation mechanism that allows maintaining the excitation balance between the two photosystems upon rapid changes in light quality/quantity. However, it has recently been shown that the association of LHCII to PSI occurs also upon long-term acclimation, and it is in fact the most common state in *A. thaliana* (Wientjes et al. 2013). In normal light conditions (100 μmol/photons/m2) around 50 % of the PSI complexes is complemented by one LHCII trimer, while this value increases in low light and decreases in high light. This allows for the simultaneous regulation of the antenna size of PSI and PSII by just changing the expression level of the two main LHCII genes (Lhcb1 and Lhcb2) Ballottari et al. 2007) in order to maintain the excitation balance between the two photosystems (Wientjes et al. 2013).

The LHCII trimer is associated with the core on the opposite side of the Lhca’s via the PsaH subunit (Lunde et al. 2000; Kouril et al. 2005). This complex is very sensitive to detergent, but it is stable in digitonian (Kouril et al. 2005; Pesaresi et al. 2009), and recently, it was purified to homogeneity (Galka et al. 2012). It was shown that the energy transfer from the LHCII trimer to the PSI core is extremely fast. Indeed, the presence of the trimer increases the antenna size of PSI by almost 25 %, while the increase in overall trapping time is only 6 ps (Wientjes et al. 2013), which indicates that there is a very good connection between the outer antenna and the core.

In summary, in most conditions, the PSI supercomplexes also bind one LHCII trimer in addition to the four Lhca’s. EET from LHCII to PSI core is extremely fast, making LHCII a perfect light harvester for the system.

#### The PSI-LHCI complex of green algae

In recent years, the study of the PSI-LHCI supercomplex has been extended to organisms other than higher plants, revealing differences in the number and organization of the antenna complexes. An overview of the PSI antennae in the different organisms can be found in Busch and Hippler (2011). It seems that in mosses, green and red algae PSI-LHCI complexes with different antenna sizes are present. In the green alga *Chlamydomonas reinhardtii* there are nine Lhca genes (Elrad and Grossman 2004), and the largest purified supercomplex contains nine Lhca subunits per core (Drop et al. 2011) although smaller complexes have also been purified (Stauber et al. 2009). The additional (when compared to plants) 5 Lhca’s form a second outer half ring around the core that is connected to the core via the 4 Lhca’s forming the inner ring (Drop et al. 2011). The larger size of PSI of *C. reinhardtii* increases its light-absorption capacity but also slows down the excitation trapping. However, the fluorescence emission at low temperature peaks around 715 nm, which is 20 nm blue-shifted as compared to that of plant PSI (Bassi et al. 1992; Germano et al. 2002). Therefore, *C. reinhardtii* PSI contains red forms that on average are at higher energies than the ones in plants (Gibasiewicz et al. 2005b), and this speeds up the trapping process. In vitro reconstitution of the 9 Lhca’s of *C. reinhardtii* has indicated that Lhca2, 4, and 9 are the antenna complexes that contain red pigments (Mozzo et al. 2010), but the exact number of red pigments in PSI of this alga is not known. Energy transfer and trapping in *C. reinhardtii* PSI-LHCI were investigated by two groups (Melkozernov et al. 2004; Ihalainen et al. 2005c). The results differ substantially, especially concerning the long decay component. This leads to different interpretations regarding the connection of the antenna with the core. However, considering the relative instability of the connection of part of the antenna to the supercomplex (Drop et al. 2011), it is possible that the sample properties were not the same in two studies.

In conclusion, PSI-LHCI is not only present in plants, but the antenna size and organization of the various complexes seem to vary for different organisms.

## What next?

Many issues regarding energy transfer and trapping in PSI still need to be fully elucidated. This is mainly due to the high complexity of the system (the core alone contains around 100 Chls), which still represents a great challenge for modeling. In this respect an additional complication is represented by the red forms, which originate from excitonically coupled pigments but also have a strong charge-transfer character. Up to now the properties of these forms could not be reproduced in silico, thus limiting the possibility to study their properties and their effect on the kinetics via modeling.

Practically all studies addressing light-harvesting in PSI-LHCI have focused on the complex of higher plants with a few exceptions dealing with the complex from *Chlamydomonas reinhardtii*. However, the analysis of new organisms indicates that many different PSI-LHCI complexes exist in nature, varying in the number of antenna complexes and it their spectroscopic properties. This variability seems to be much more pronounced than in the case of PSII where LHCII trimers with properties similar to those of higher plants have been observed in many organisms, suggesting that the antenna complexes of PSI play a role in adaptation. This variability, on the other hand, provides the possibility to compare the functional behavior of PSI complexes which differ in antenna size and energy, in order to determine the robustness of the complex.

The comparison of all these complexes and of the environmental conditions in which these host organisms live would help in answering a long-standing question: what is the role of the red forms? Although we nowadays know a lot about their origin and their effect on the excitation trapping, we cannot answer this fundamental question yet. The possibility to produce plants or algae lacking red forms and to compare their growing capacity and their performance with those of the corresponding WT will form another strategy to unravel their physiological function. In principle, this is feasible because in vitro mutagenesis has clearly indicated which residues need to be changed to shift the red absorption of Lhca’s to the blue.

Finally, in most organisms, the antenna of PSI is not only composed of Lhca, but also of LHCII. Although the PSI-LHCI-LHCII complex of higher plants has now been studied in some detail, very little information is available regarding this complex in other organisms. The case of *Chlamydomonas reinhardtii* is particularly interesting as it is generally believed that most of the LHCII moves to PSI in state 2. However, what does the PSI-LHCI-LHCII complex of *C. reinhardtii* look like and how is this large number of LHCII’s associated with PSI? And finally, how efficient is the trapping in these large PSI-LHCI-LHCII supercomplexes?
